# Recruitment and retention of women in fishing communities in HIV prevention research

**DOI:** 10.11604/pamj.2015.21.104.4962

**Published:** 2015-06-09

**Authors:** Ali Ssetaala, Jessica Nakiyingi-Miiro, Stephen Asiimwe, Annet Nanvubya, Juliet Mpendo, Gershim Asiki, Leslie Nielsen, Noah Kiwanuka, Janet Seeley, Anatoli Kamali, Pontiano Kaleebu

**Affiliations:** 1 UVRI-IAVI HIV Vaccine Program, Entebbe, Uganda; 2 MRC/UVRI Uganda Research Unit on AIDS, Uganda; 3 University of Georgia, Athens, GA, USA; 4 Kabwohe Clinical Reserach Center(KCRC), Kabwohe, Uganda; 5 International AIDS Vaccine Initiative (IAVI), New York, USA; 6 Makerere College of Health Sciences, School of Public Health, Makerere, Kampala, Uganda

**Keywords:** Women, fishing communities, recruitment, retention, HIV, prevention, research

## Abstract

**Introduction:**

Women in fishing communities in Uganda are more at risk and have higher rates of HIV infection. Socio-cultural gender norms, limited access to health information and services, economic disempowerment, sexual abuse and their biological susceptibility make women more at risk of infection. There is need to design interventions that cater for women's vulnerability. We explore factors affecting recruitment and retention of women from fishing communities in HIV prevention research.

**Methods:**

An HIV incidence cohort screened 2074 volunteers (1057 men and 1017 women) aged 13-49 years from 5 fishing communities along Lake Victoria using demographic, medical history, risk behaviour assessment questionnaires.1000 HIV negative high risk volunteers were enrolled and followed every 6 months for 18 months. Factors associated with completion of study visits among women were analyzed using multivariable logistic regression.

**Results:**

Women constituted 1,017(49%) of those screened, and 449(45%) of those enrolled with a median (IQR) age of 27 (22-33) years. Main reasons for non-enrolment were HIV infection (33.9%) and reported low risk behaviour (37.5%). A total of 382 (74%) women and 332 (69%) men completed all follow up visits. Older women (>24 yrs) and those unemployed, who had lived in the community for 5 years or more, were more likely to complete all study visits.

**Conclusion:**

Women had better retention rates than men at 18 months. Strategies for recruiting and retaining younger women and those who have stayed for less than 5 years need to be developed for improved retention of women in fishing communities in HIV prevention and research Programs.

## Introduction

Fishing communities (FCs) in Uganda are still among key populations for HIV/AIDS spread despite first noticing HIV more than two decades ago in a fishing community [1]. FCs along Lake Victoria have an HIV prevalence of 22-29%, with an incidence of 3.4 -5 per 100 person years at risk (PYAR) [2–5].

Women in fishing communities are disproportionately affected by HIV/AIDS with a prevalence of 33-34% and incidence of 3.4-4.5/100PYAR, more than four times the national averages [2–6]. Women′s livelihoods are threatened by the high HIV rates, which may also lead to resurgence of the disease beyond fishing communities. Women in fishing communities are particularly vulnerable to HIV infection as current prevention interventions in these communities may not readily accessible to women in fishing communities. Abstinence in the presence of economic vulnerability may be hard to practice. Faithfulness needs participation of both partners. Condoms in fishing communities may not be accessible, and male partners may not be willing to use them as condom use may be perceived as lack of trust.

It is important for women from fishing communities to participate in HIV prevention research for the design of prevention interventions that suit them. Social, economic, cultural, biological factors that facilitate or hinder recruitment and retention should be taken into account when considering women's participation. Factors that may affect women's recruitment in HIV prevention research and vulnerability to HIV/AIDS include; Socio-cultural norms regarding sex, with women being expected to be chaste, subservient partners, making it hard for women in fishing communities to participate in HIV research and be fully informed of safer sex and reproductive health, let alone negotiating safer sex [7, 8].

Motherhood, a social expectation of women which makes contraceptive use and protected sex challenging for women without children even those with fewer children [9]. HIV prevention research that requires use of contraception may be hard for women in fishing communities for fear of being labelled social misfits in a society that values fertility. Women with children may fail to participate in HIV prevention research that seems to risk their safety as mothers and the safety of their children [10, 11].

African culture values the head of household (usually a man) as the final decision maker, including decisions that directly affect the wife's health [12]. This hinders women's participation in research as they have to seek informal consent from the head of the household or even withdraw consent if the head wasn't aware of her participation at study commencement. Fishing and related tasks are most times gender specific, with the more paying roles being dominated by men. This may encourage HIV risk activities like transactional sex for economic survival and diminished ability to negotiate safer sex [13–16]. Physical violence, sexual, psychological and financial abuse of women may increase HIV vulnerability, limit access to prevention research, health information and care services. Many a woman may fail to communicate such abuses for fear of losing their spouses/partners from whom they are socio-economically subordinates [17–20]. Fisher folk dearth access to health and social services yet they are most at risk,[2, 5, 6, 14, 16, 21, 22], together with the vulnerabilities of women, this may affect participation in HIV prevention research as decisions to participate may be influenced by the expected access to health services. Given these issues studies on recruitment and retention of women in FCs in HIV prevention research should be taken as top priority to help design interventions that suit this vulnerable group including receiving suitable specific updated information and services regarding protection from HIV.

Factors associated with women's participation in research vary by community, yet little data is available on recruitment and retention of women in FCs. Successful development of future programs and research targeting women in fishing communities depends on addressing factors affecting recruitment and retention. We explore factors affecting recruitment and retention of women in FCs enrolled in a prospective HIV incidence cohort within selected Ugandan FCs along Lake Victoria. This paper is very important in providing insights into the experience of recruiting and maintaining women in FCs in cohort studies, treatment programs among this socially unique ‘mobile’ population in a resource limited setting where women are more vulnerable.

## Methods

### Recruitment and follow up of volunteers

A prospective observational 18 months cohort study recruited and followed 1,000 high risk HIV negative consenting volunteers aged 13-49 years from 5 conveniently selected (population size more than 1000 and distance to research within 50km), fishing communities along the shores of Lake Victoria during 2009-2011. Recruitment procedures have been previously described [5].

The study aimed at determining HIV prevalence, incidence, circulating HIV-1 subtypes and the socio-behavioural characteristics of fisher folk in preparation for future HIV prevention research. This analysis was performed to describe the characteristics of women who participated in the study, identify factors associated with completion of follow up visits and provide information on whether particular categories of women should be targeted to increase participation and retention in HIV prevention research among these socially unique communities.

Following mapping and census of study sites, community meetings involving community confidants like the local councils, beach management units, and opinion leaders preceded the screening process to provide information about the study. Screening for identifying eligible high risk HIV negative volunteers involved interviewer administered demographic, medical history, risk assessment (Alcohol consumption, reported STI symptoms in previous three months, unprotected sexual intercourse with more than one partner or new sexual partner in previous three months, being in a discordant relationship and volunteer or partner being away from home for at least two nights in the previous month) structured questionnaires, HCT (HIV counselling and testing) and provision of blood samples. Medical history, collection of risk factor data, symptom directed physical examinations and HIV tests were done at every 6 months follow up visit.

HIV testing was done using the Determine rapid kit (Determine; ABBOTT Laboratories, Diagnostic division, Illinois, Chicago, USA), with further confirmatory testing using enzyme linked Immunosorbent assays (Vironostika HIV Uni-Form II plus 0 microelisa system, Biomerieux, Boxtel, Netherlands and Murex HIV-1.2.0 Murex, Biotech Limited, Dartford, UK and by Western blot, Calypte Biomedical Corporation, Rockville, Maryland if indeterminate).

Detailed locator information was obtained and updated during the 6 monthly visits. Home visits and telephone reminders were done for following up volunteers. Some Volunteers who were compliant with study visits were trained as participant leaders. These helped remind others to attend their visits and provide reports on volunteers who have migrated out of the study area. Information about missed visits was regularly generated from the data base and passed on to the field team to trace the volunteers. Missed visits and reasons were documented whenever they occurred. Volunteers were helped with appropriate written referrals to HIV/AIDS care and other health services for those who needed them.

### Ethics

Uganda Virus Research Institute Science and Ethics Committee and the Uganda National Council of Science and Technology provided ethical approvals for this study. Informed consent and assent were sought from all participants before enrolment.

### Statistical analysis

Data were entered using MS Access 2003 (1992–2003 Microsoft Corporation, Redmond, Washington), and analyzed using Stata/SE11 (1996–2011 StataCorp, College Station, Texas, USA). The major aim was to identify factors associated with the dichotomous outcome; retention of women in this cohort.

Baseline characteristics of the women were analyzed by univariate and bivariate analysis using chi-square tests or fisher's exact tests. Stratification of some variables and cross tabulations was also done. Multivariable regression analysis was done to determine the effects of variables on the outcome. Covariates selected for the univariate analysis were age (13-24, 25-29 and 30 or more years), ethnicity (Baganda/non Baganda), religion (catholic, Anglican, other Christian, and Muslim), education(complete primary, incomplete primary, none), marital status(married/not married),financial dependants(yes/no), occupation (fishing& related tasks, bar workers, business/trade, farming, other jobs and not earning) and duration of stay in the community( less than five years/more than five years).

Multivariable analysis was done using forward stepwise logistic regression, with factors significantly associated with completion of all study visits at p-values ≤ 0.05 remaining in the final model. Covariates included in multivariable analysis were based on bivariate statistical significance at an alpha of <0.10 and biological plausibility. The final model included agegroup, religion,duration of community stay, occupation and marital status.

Retention at each follow up visit was based on the proportion of women who returned for all the three 6 monthly follow up visits, including person time contributed by those who tested HIV positive (sero-converted) and the proportion of women who returned for at least one follow up visit.

## Results

### Socio-demographic characteristics of women in fishing communities

A total of 449 women were enrolled in the cohort with a median, inter quartile range (IQR) age of 27 (22-33) years. Majority of women worked in fishing related tasks (41.2%), some worked in bars (13.6%), farming (13.1%) and business (12%) (p < 0.001). Many women were married (73.5%), did not complete primary education (41.9%), had financial dependents (63.9%), were Catholics (41.0%), and had lived in the community for <5 years (66.1%) (See Table 1).

**Table 1:** Baseline demographic characteristics of women in fishing communities

Variable	Women		Men	
	No.	%	No.	%
**Age**				**p = 0.06** ^**+**^
13-24 yrs	204	45.4	209	37.9
25-29 yrs	97	21.6	138	25.1
30+ yrs	148	33.0	204	37.0
**Education level**				**p = 0.7**
Completed Primary	211	47.0	268	48.6
Incomplete primary	188	41.9	229	41.6
None	50	11.1	54	9.8
**Marital status**				**p < 0.001** ^**++**^
Not married	119	26.5	205	37.2
Married	330	73.5	346	62.8
**Occupation**				**p < 0.001** ^**++**^
Fishing & related tasks	185	41.2	435	79.0
Bar workers	61	13.6	2	0.4
Business/trade	54	12.0	36	6.5
Farming	59	13.1	27	4.9
Other jobs	29	6.5	45	8.2
**Religion**				**p = 0.04** ^**++**^
Catholic	184	41.0	265	48.1
Anglican	106	23.6	133	24.1
Other Christian	47	10.5	37	6.7
Muslim	112	24.9	116	21.1
**Financial dependents**				**p = 0.002** ^**++**^
No	161	36.1	149	27.1
Yes	285	63.9	400	72.9
**Duration in community**				**p < 0.001** ^**++**^
5-45 yrs	150	33.9	245	45.0
<5 yrs	292	66.1	300	55.1

++ p-value < = 0.05,

+ 0.05 < p-value < 0.1

### Women recruitment

Recruitment from the five fishing communities was done over 6 months, with 1, 017 women screened to enrol 449 (Screening: enrolment of 2.3:1). Fewer women were enrolled; 44.9% (95%CI = 41.1, 47.2) women vs 55.1% (49.1, 55.1) men, Odds Ratio (OR) 1.38 (1.16, 1.64), p < 0.001. The main reasons for non-enrolment into the HIV negative cohort were HIV infection, and reported low risk of HIV. Women were more likely to be HIV infected as prevalence was higher among women (33.9%) than men (23.9%) [2]. Women were less likely not to be enrolled because they were not at risk of HIV infection (37.5% (95%CI= 33.5, 41.5)) than men (47.1% (95%CI = 42.6, 51.5)). OR for not at risk 1.48 (1.16, 1.89), p = 0.002.

### Women's retention

Although fewer women were enrolled, 93.3% returned for at least one follow-up visit over the 18 months compared to 90.7% men. Including person time contributed by those who sero-converted, more women attended all three 6monthly study visits (73.9% (95%CI 69.9, 78)) than men (69.3% (65.5, 73.2)), p = 0.032. Factors that were associated with better retention (completion of all study follow up visits, including sero-conversion) among women in fishing communities were age over 24 years, staying in the community for at least 5 years and not earning an income.

Younger women aged 13-24 years were less likely to attend all visits compared to older women aged 30+ years and those who had been in the community for less than 5 years were less likely to attend all visits. Older women (30+ years) had not spent two or more nights away from home in the previous month compared to younger women (OR = 2.4 (95% CI 1.5, 4)). Occupation was not significantly associated with attendance at all visits, although women working in bars or the hair dressers, or those with professional jobs had lower attendance rates at all visits compared to those with other occupations (See Table 2).

**Table 2:** Multivariable analysis of study completion by women in fishing communities

Variable	No.	Those who completed			Crude analysis				Multivariable analysis			
		%	95%CI		Odds ratio	95%CI		p-values	Odds ratio	95%CI		p-value
All women	449	73.9	69.9	78.0				0.108 ^1^				p < 0.001^++2^
**Age-group**												
13-24 yrs	204	67.2	60.7	73.7					Ref			
25-29 yrs	97	77.3	68.8	85.8	1.7	1.0	2.9	0.073^+^	1.9	1.0	3.5	0.043^++^
30+ yrs	148	81.1	74.7	87.5	2.1	1.3	3.5	0.004^++^	2.2	1.2	3.9	0.009^++^
**Religion**												
Catholic	184	79.3	73.4	85.3	1.7	1.0	3.0	0.047^++^	1.7	1.0	3.0	0.068^+^
Anglican	106	68.9	59.9	77.8					Ref			
Other	47	66.0	51.9	80.0	0.9	0.4	1.8	0.72	0.9	0.4	1.9	0.75
Muslim	112	73.2	64.9	81.5	1.2	0.7	2.2	0.48	1.4	0.8	2.7	0.27
**Duration in community**												
5-45 yrs	150	84.7	78.8	90.5					2.1	1.2	3.6	0.007^++^
<5 yrs	292	68.5	63.1	73.9	2.5	1.5	4.2	p < 0.001^++^	Ref			
**Occupation**												
Fishing & related tasks	185	77.3	71.2	83.4	1.9	1.0	3.6	0.041^++^	1.9	1.0	3.8	0.063^+^
Bar workers	61	63.9	51.5	76.3					Ref			
Business/trade	54	75.9	64.1	87.7	1.8	0.8	4.0	0.17	1.8	0.8	4.3	0.19
Farming	59	72.9	61.2	84.6	1.5	0.7	3.3	0.29	1.2	0.5	2.9	0.63
Other jobs	29	65.5	47.1	83.9	1.1	0.4	2.7	0.88	1.1	0.4	3.1	0.81
Not earning	61	77.0	66.2	87.9	1.9	0.9	4.2	0.12	2.4	1.0	5.7	0.048^++^
**Marital status**												
Single	43	72.1	58.1	86.1	1.3	0.6	2.9	0.57	2.3	0.9	5.8	0.069^+^
Married	330	75.8	71.1	80.4	1.5	0.9	2.6	0.12	1.6	0.9	3.0	0.11
Divorced/Widowed	76	67.1	56.3	77.9					Ref			

++ P-value < = 0.05,

+ 0.05 < p-value < 0.1.

1 p-value comparing completion rates between males and females.

2 p-values from the multivariable analysis. Not earning occupation were participants who did activities that never involved any form of income

## Discussion

Less than half (45%) of the cohort participants were women with the main reasons for none enrolment being HIV infection (prevalence was higher among women (33.9% vs 23.9%) [2] and reported low HIV risk behavior. Women were more readily available for follow up visits, as 93% came back for at least one follow up visit and 74% successfully completed all three scheduled visits. Retention was associated with older age, more than 24 years, staying in the community for at least 5 years and not earning an income. The HIV incidence cohort recruitment was conducted among HIV high risk participants enrolled from a cross sectional survey following mapping and census of the 5 fishing communities [5], which is strength for the study. Our study had some limitations: Use of face to face interviews may have led to under reporting of socially undesirable risk behaviour [23].

Participants were not specifically asked why they decided to participate in the research, why they decided to come for study visits and reasons for missing study visits or dropping out, which could have provided more information on recruitment and retention.

Fewer women were recruited into the cohort probably due to a higher HIV prevalence among women compared to men [2], which is similar to another study [24], though less women reported HIV risk behaviours like STIs symptoms in the past three months, inconsistent condom use and being in discordant relationships which increase vulnerability to HIV infection [2]. This less reporting of HIV risk behaviours is in conformity with socio-cultural constructs where expectations and norms dictate that chaste women should be ignorant about sex, unmarried young women should be virgins and play a passive role in discussions of sex and sexual partners [9, 25]. Such socio-cultural expectations, women's reliance on men's income/ fish catch makes it hard to negotiate safe sex or even leave risky relationships when they have no alternative sources of income [16, 25], coupled with limited access to health information and services may lead to fisher folk women under reporting HIV risk sexual behaviour for fear of being perceived as social misfits or promiscuous. This may have led to fewer women in fishing communities being recruited into the HIV negative high risk cohort as some reported contrary to their actual behaviour and others needed consent from their spouses or sexual partners to be recruited.

Inadequate education (42% didn't complete primary level) leaves women in FCs at a lower social status with jobs like fish smoking, drying fish, working as restaurant attendants or bar maids, which put them at increased risk of sexual exploitation. Education was not significantly associated with retention of women in this study, though another study in Malawi showed that lower education level was associated with loss to follow up [26].

Cohort retention rates for women in fishing communities were better than those of men, probably because fewer women were engaged in actual fishing which requires more time away from home, hence were readily available within their communities and homes [2]. Older women, more than 30 years were more likely to attend all study visits as they were more settled, spending fewer nights away from their homes in the previous month. These findings are similar to other studies done in rural communities [27]. However younger women (<24 years) who were less likely to return for follow up visits were also found to have a higher HIV incidence in other studies [4, 5, 28], implying that efforts towards retaining younger women in HIV prevention research and other prevention programs are needed in these fishing communities.

Most women in fishing communities were married with financial dependants, which too explain the higher retention rates and likelihood of attendance of all scheduled study visits as married women tend to have more social support. Being unmarried is a risk factor for STIs including HIV [28], due to increased number of sexual partners, diminished ability to negotiate safer sex and limited health awareness [7, 8, 14, 15]. Women without income were better retained in the cohort because they were more readily available to comply with study visits. Access to health care provided during the study could also have been motivating factor as women without income might have been finding it harder to access better health services due to financial limitations [7, 29].

None income earners might have been also interested in the little monetary compensation for time spent at each of the follow up visits. Income earning women were less likely to attend all study visits probably because the reimbursements for time spent were not sufficient for them to leave their work schedules and come for study visits. Similar studies did not find an association between employment and study retention [27, 28].

Having stayed in the fishing communities for at least five years was associated with better cohort retention as women in fishing communities who have lived in the communities longer are less likely to migrate and have over time established social support net works and can easily be located by other community members.

Women who were new immigrants (<5 years in the community), who were less likely to return for follow up visits were found to have a higher HIV incidence in another study [5]. This is similar to another study where migrants engaged in more higher risk sexual behaviours [8]. This may imply that HIV prevention research targeting high incidence groups in FCs may need to devise innovative ways of retaining new immigrants in such studies.

Our study means that women in FCs can participate in HIV prevention research with favourable retention rates, though research on how to recruit and retain young women (<24 years) who have stayed in communities for less than five years into HIV prevention research needs to be conducted. This will help design interventions that carter for women's vulnerability in FCs along Lake Victoria.

## Conclusion

Women in fishing can be recruited in HIV prevention research with favourable retention rates. There is need to design strategies to make retention rates better especially for young women. This will ensure optimization of HIV preventive research and care for women in FCs.
